# Fetal cardiovascular response to acute hypoxia during maternal anesthesia

**DOI:** 10.14814/phy2.14365

**Published:** 2020-02-05

**Authors:** Tamara J. Varcoe, Jack R. T. Darby, Stacey L. Holman, Emma L. Bradshaw, Tim Kuchel, Lewis Vaughan, Michael Seed, Michael D. Wiese, Janna L. Morrison

**Affiliations:** ^1^ Early Origins of Adult Health Research Group University of South Australia Adelaide Australia; ^2^ School of Pharmacy and Medical Sciences University of South Australia Adelaide Australia; ^3^ South Australian Health and Medical Research Institute Adelaide Australia; ^4^ Hospital for Sick Children Toronto Canada

**Keywords:** acute hypoxia, anesthesia, blood pressure, fetus

## Abstract

Preclinical imaging studies of fetal hemodynamics require anesthesia to immobilize the animal. This may induce cardiovascular depression and confound measures under investigation. We compared the impact of four anesthetic regimes upon maternal and fetal blood gas and hemodynamics during baseline periods of normoxia, and in response to an acute hypoxic challenge in pregnant sheep. Merino ewes were surgically prepared with maternal and fetal vascular catheters and a fetal femoral artery flow probe at 105–109 days gestation. At 110–120 days gestation, ewes were anesthetized with either isoflurane (1.6%), isoflurane (0.8%) plus ketamine (3.6 mg·kg^−1^·h^−1^), ketamine (12.6 mg·kg^−1^·h^−1^) plus midazolam (0.78 mg·kg^−1^·h^−1^), propofol (30 mg·kg^−1^·h^−1^), or remained conscious. Following 60 min of baseline recording, nitrogen was administered directly into the maternal trachea to displace oxygen and induce maternal and thus fetal hypoxemia. During normoxia, maternal PaO_2_ was ~30 mmHg lower in anesthetized ewes compared to conscious controls, regardless of the type of anesthesia (*p* < .001). There was no effect of anesthesia on fetal mean arterial blood pressure (MAP; *p* > .05), but heart rate was 32 ± 8 bpm lower in fetuses from ewes administered isoflurane (*p* = .044). During maternal hypoxia, fetal MAP increased, and peripheral blood flow decreased in all fetuses except those administered propofol (*p* < .05). Unexpectedly, hypoxemia also induced fetal tachycardia regardless of the anesthetic regime (*p* < .05). These results indicate that despite maternal anesthesia, the fetus can mount a cardiovascular response to acute hypoxia by increasing blood pressure and reducing peripheral blood flow, although the heart rate response may differ from when no anesthesia is present.

## Graphical abstract

Preclinical imaging studies of fetal haemodynamics require anaesthesia to immobilise the animal. In the presence of isoflurane, isoflurane/ketamine and ketamine/midazolam, but not propofol anaesthesia, maternal and fetal hypoxaemia increased fetal mean arterial pressure and decreased peripheral blood flow. Acute fetal hypoxaemia induced fetal tachycardia regardless of anaesthetic type. These results have implications for the design and interpretation of preclinical imaging studies where anaesthesia is required.

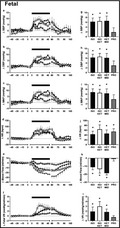

## 1 INTRODUCTION

Acquisition of high‐resolution magnetic resonance images (MRI) of the fetus *in utero* has the potential to expand our understanding of fetal hemodynamics during normal and abnormal development. Preclinical studies in a relevant animal model are required to develop and validate these technologies. Due to their size, fetal developmental milestones, low rate of multiple fetuses, and resilience to surgical implantation of fetal vascular catheters, the pregnant ewe is an excellent animal model of human pregnancy (Morrison et al., 2018). Ewes cannot be expected to remain still throughout an MRI session, and thus anesthesia is required to protect their well‐being and enable collection of data. However, anesthetic agents can induce cardiovascular depression that may confound measures obtained by MRI (Khan, Hayes, & Buggy, 2014, 2014; Saraswat, 2015). Therefore, prior to the conduct of preclinical studies using these technologies, an assessment of the maternal and fetal cardiovascular response to a variety of anesthetic agents is required to provide evidence for selection of the optimum anesthetic protocol.

There are many anesthetic agents and combinations that are routinely used in veterinary medicine for which the chemical, physical, and pharmacological properties differ greatly. Isoflurane is an inhalation anesthetic that acts upon the α1‐subunit of the GABA_A_ receptor to enhance responsiveness to endogenous GABA, thereby prolonging the duration of postsynaptic inhibition (Brohan & Goudra, 2017). Alternatively, the lipophilic anesthetic propofol can be administered by intravenous infusion. While this drug also acts to enhance the actions of GABA, it has been shown to interact with a variety of neurotransmitter receptors including the ligand‐gated ion channel superfamily, G protein‐coupled receptors, and voltage dependent ion channels (Trapani, Altomare, Liso, & Sanna, 2000). The benzodiazepine midazolam potentiates the effects of GABA by binding to sites upon the GABA_A_ receptor (Olkkola & Ahonen, 2008), but alone does not induce the same degree of neuronal inhibition as isoflurane or propofol. However, midazolam can be administered in conjunction with ketamine, which acts to suppress excitatory synaptic activity by blockade of *N*‐methyl‐d‐aspartate (NMDA) receptors (Sleigh, Harvey, Voss, & Denny, 2014).

When administered to adults, both isoflurane and propofol decrease cardiac output, systemic vascular resistance, and mean arterial pressure, and result in a baroreceptor‐mediated increase in heart rate (Khan et al., 2014, 2014). Alternatively, ketamine, despite its direct negative cardiac inotropic effect, increases cardiac output, systemic vascular resistance, mean arterial pressure, and heart rate due to increased sympathetic outflow (Berry, 2007; Khan et al., 2014). The impact of these agents upon the fetal cardiovascular system is less clear but may be mediated by both direct effects upon the fetus itself, or indirectly following anesthesia‐induced maternal cardiorespiratory depression.

Development within a relatively hypoxic environment has prompted fetal adaptations that ensure sufficient oxygen is available to meet the metabolic demands necessary for growth and development. However, the fetus can respond to periods of acute hypoxia, for example, that may occur during uterine contractions or transient umbilical cord compressions, through the activation of carotid chemoreceptors that lead to dominant vagal nerve stimulation of the fetal heart and consequent bradycardia (Boddy, Dawes, Fisher, Pinter, & Robinson, 1974; Cohn, Sacks, Heymann, & Rudolph, 1974; Giussani, Spencer, Moore, Bennet, & Hanson, 1993; Raff & Wood, 1992; Wassink et al., 2007). The decreased fetal heart rate in turn slows the passage of blood through the fetal circulation, permitting increased efficiency of gaseous exchange in vascular beds (Boudoulas, Rittgers, Lewis, Leier, & Weissler, 1979; Gardner, Giussani, & Fowden, 2003). Simultaneously, increased sympathetic outflow to the periphery increases vasoconstriction and reduces peripheral blood flow, thereby ensuring maintenance of oxygenated blood flow to the fetal brain, heart, and adrenals at the expense of peripheral tissues (Bennet, 2017; Giussani, 2016) reviewedin. Given the maternal cardiorespiratory depression that occurs in response to general anesthesia, the effect of anesthetic exposure on fetal hemodynamics under both normoxia and in response to acute hypoxia may differ.

The aim of this study was to identify an anesthetic regime that provides appropriate anesthesia for the ewe and allows the fetus to remain capable of mounting a cardiovascular response to hypoxia. We compared the impact of four different anesthetics on maternal and fetal hemodynamics during baseline periods of normoxia and upon the fetal response to an acute hypoxic challenge in pregnant sheep.

## 2 METHODS

### 2.1 Ethical considerations

All experiments were conducted in accordance with the ARRIVE guidelines (Kilkenny, Browne, Cuthill, Emerson, & Altman, 2010) and Australian Code of Practice for the Care and Use of Animals for Scientific Purposes (National Health & Medical Research Council, 2013), with approval from the South Australian Health and Medical Research Institute (SAHMRI) Animal Ethics Committee. All investigators understood and followed the ethical principles outlined by Grundy (2015).

### 2.2 Animals and surgery

Thirteen pregnant Merino ewes bearing a singleton fetus (94–98 days gestation, term 150 ± 3 days) were transported from SAHMRI farm at Burra, South Australia, to the Preclinical Imaging and Research Laboratories, Gilles Plains, South Australia, where they were housed individually in a 12‐hr light–dark photoperiod with ad libitum access to water. Ewes were fed a daily ration of 1 kg lucerne chaff (85% dry matter, metabolizable energy content = 8.3 MJ·kg^−1^) supplemented with 300 g pellets containing straw, cereal, hay, clover, barley, oats, lupins, almond shells, oat husks, and limestone (89% dry matter, metabolizable energy content = 11.6 MJ·kg^−1^, Ridley Agriproducts Sheep Nutrition Ration, Murray Bridge, South Australia, Australia).

### 2.3 Catheterization surgery

At 105–109 days of gestation (maternal weight: 65.1 ± 2.2 kg, fetal sex: 8 males, 5 females), anesthesia was induced with diazepam (0.3 mg·kg^−1^, Lyppards, Melbourne, Australia) followed by ketamine (7 mg·kg^−1^, Lyppards, Melbourne, Australia), then maintained with 1.5%–2.5% isoflurane (Fluothane, ICI, Melbourne, Australia) in oxygen. Vascular catheters were implanted in the maternal carotid artery and jugular vein, fetal femoral artery and vein, as well as the amniotic cavity as previously described (Danielson, McMillen, Dyer, & Morrison, 2005). Transonic flow probes were placed on the fetal femoral artery (Morrison, Chien, Riggs, Gruber, & Rurak, 2002; Poudel, McMillen, Dunn, Zhang, & Morrison, 2015). Fetal catheters and flow probes were exteriorized through an incision in the ewe's flank. A catheter (OD = 2.7 mm, ID = 1.5 mm) was inserted 2 cm into the maternal trachea (Wood et al., 2013). At surgery, antibiotics were administered intramuscularly to the ewe (3.5 ml of 150 mg·ml^−1^ procaine penicillin, 112.5 mg·ml^−1^ benzathine penicillin, plus 2 ml of 125 mg·ml^−1^ dihydrostreptomycin, Lyppards, Melbourne, Australia) and fetus (1 ml of 150 mg·ml^−1^ procaine penicillin, 112.5 mg·ml^−1^ benzathine penicillin, plus 1 ml of 125 mg·ml^−1^ dihydrostreptomycin). Antibiotics were administered intramuscularly to each ewe for 3 days after surgery and intra‐amniotically (500 mg Ampicillin, Clifford Hallam Healthcare, Adelaide, Australia) for 4 days after surgery. Analgesia was administered subcutaneously at 16:00 hr on the day prior to surgery and repeated 24 hr later (meloxicam, subcutaneous, 0.5 mg·ml^−1^, Lyppards, Melbourne, Australia (Varcoe et al., 2019). Clinical record sheets were completed daily to monitor behavior (appetite, thirst, defecation, urination, and alertness) and fetal arterial blood collected daily for blood gas analysis. Ewes were allowed to recover from surgery for at least 3 days before undergoing experimental interventions.

### 2.4 Experimental interventions

Ewes underwent anesthesia protocols on up to three separate occasions over a 10‐day period (between 110 and 120 days gestation, average = 115 ± 3 days), with at least 3 days between each intervention. The order of the anesthetic agents was determined using random number generation. Following induction, ewes were intubated and ventilated with room air to avoid hyperoxygenation of both ewe and fetus. The anesthesia interventions consisted of four commonly used anesthetic regimes used in veterinary practice for large animals (Flecknell, 2015; Riebold, 2015):

1. Isoflurane (ISO): Anesthesia‐induced with diazepam (0.3 mg·kg^−1^) and ketamine (7 mg·kg^−1^), then maintained with isoflurane (mean = 1.6%, range = 0.8%–3.0%), *n* = 7.
2. Isoflurane and Ketamine (ISO/KET): Anesthesia‐induced with diazepam (0.3 mg·kg^−1^) and ketamine (7 mg·kg^−1^), then maintained with continuous intravenous infusion of ketamine (3.6 mg·kg^−1^·h^−1^) and isoflurane (mean = 0.8%, range = 0.5%–2%), *n* = 6.
3. Ketamine and Midazolam (KET/MID): Anesthesia‐induced with 5 mg·kg^−1^ ketamine and 0.5 mg·kg^−1^ midazolam (Lyppards, Melbourne, Australia), then maintained with continuous intravenous infusion of ketamine (mean = 12.6, range = 11.5–13.8 mg·kg^−1^·h^−1^) and midazolam (mean = 0.78, range = 0.75–0.90 mg·kg^−1^·h^−1^), *n* = 7.
4. Propofol (PRO): Anesthesia‐induced with propofol (5 mg·kg^−1^, Lyppards, Melbourne, Australia), then maintained with continuous intravenous infusion (mean = 29.9, range = 22–38 mg·kg^−1^·h^−1^), *n* = 8.
5. Conscious (CON): Ewes were separately assessed while conscious and individually housed, *n* = 9.

### 2.5 Experimental protocol

Following induction of anesthesia at approximately 09:00 hr, ewes were placed on their left side on a surgical table and connected to physiological monitoring equipment including pulse oximeter and capnograph (Philips IntelliVue MP5 or MP30, North Ryde, NSW, Australia), displacement transducers, and flowmeter (TS402, Transonic). The plane of anesthesia was monitored by trained animal technicians whom were not members of the research team by palpebral reflex and pedal withdrawal every 5 min and rated on a scale of 0 to 3 (no response, slight, moderate, and extreme withdrawal), and drug administration was adjusted to maintain a level of anesthesia of 1 throughout the protocol. Maternal body temperature, blood pressure (BP), heart rate (HR), oxygen saturation (SaO_2_), and end tidal (ET) CO_2_ were recorded every 5 min. Respiratory rate was manipulated to ensure ETCO_2_ remained below 45 mmHg.

**Figure 1:**
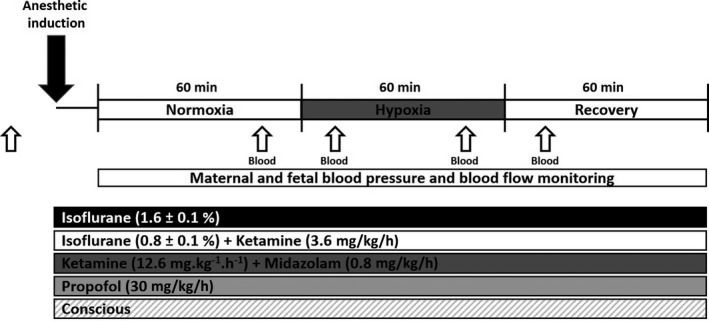
Timeline of anesthetic administration, hypoxia, and hemodynamic assessments. Details of induction, ventilation, depth of anesthesia, pulse oximetry, and ETCO_2_ monitoring are described within the methods. Open arrow represents timing of blood sampling for blood gas analysis

Following 60 min of baseline recording, acute hypoxia was induced by displacement of oxygen with nitrogen into the maternal trachea via either the tracheal catheter or the ventilator (Figure 1). The level of nitrogen flow was adjusted to maintain a level of maternal oxygen saturation between 60% and 70%, as assessed by pulse oximetry every 5 min. Maternal hypoxia was maintained for at least 45 min, followed by 60 min of recovery. Ewes were administered 1 L of saline (IV) throughout the period of anesthesia. Following completion of all experimental interventions, ewes were humanely killed by overdose with pentobarbital (100 mg kg^−1^, Lethabarb, Lyppards, Melbourne, Australia).

### 2.6 Analysis of arterial blood pressure, blood flow, and heart rate data

The flow probe was connected to a Transonic flow meter and all cardiovascular variables were sampled at a rate of 400 Hz using Chart 7 (ADInstruments, Castle Hill, Australia), and arterial pressure was corrected by subtracting intra‐amniotic pressure. Fetal systolic blood pressure (SBP) and diastolic blood pressure (DBP) were calculated as the mean maximum and minimum pressure, respectively. Fetal mean arterial pressure (MAP) was calculated using the formula: MAP (mmHg) = DBP + 0.4(SBP − DBP) (Morrison, Carmichael, Homan, & Richardson, 1997). Maternal MAP was calculated using the formula 0.33(SBP) + 0.66(DBP). Vascular resistance (VR) was calculated by dividing MAP by femoral blood flow (FBF). HR was derived from the BP and flow signals for cross checking. All data were analyzed in 1‐min nonoverlapping blocks.

### 2.7 Arterial blood gas measurements

Blood (1 ml) was collected from the maternal carotid artery and fetal femoral artery 15 min prior to induction of hypoxia, 15 and 45 min following the commencement of hypoxia, and 15 min following recovery (Figure 1). Samples were analyzed for PO_2_, PCO_2_, pH, oxygen saturation (SO_2_), and hemoglobin (Hb) on an ABL80 or ABL90 blood gas analyzer (Radiometer, Copenhagen, Denmark).

### 2.8 Statistical analyses

Data were analyzed using SPSS v25 (IBM Corp., Armonk, NY, USA). To compare the plane of anesthesia achieved under each anesthetic regimen, pedal withdrawal, and palpebral reflex data throughout the period of recording was averaged and analyzed by ANOVA. To assess the impact of anesthesia during normoxia, the values obtained from maternal and fetal SBP, DBP, MAP, heart rate, and blood flow during the hour prior to nitrogen exposure were averaged and included in a linear mixed model to assess effects of treatment (ISO, ISO/KET, KET/MID, PRO, and CON), with ewe ID as a random factor. To assess the impact of anesthesia upon the response to hypoxia, maternal and fetal blood pressure, heart rate, blood flow, and vascular resistance were included in a linear mixed model to assess effects of treatment (ISO, ISO/KET, KET/MID, and PRO) and condition (average normoxia and average hypoxia) as a repeated measure, with ewe ID as a random factor. Only animals for which maternal hypoxemia was achieved were included in these analyses (i.e., maternal pulse oximetry SaO_2_ dropped and remained below 75% throughout the period of nitrogen exposure). The effect of treatment (ISO, ISO/KET, KET/MID, PRO, and CON) and time (−15 min, +15 min, +45 min, and recovery) on blood gas was determined via repeated measures linear mixed model with ewe ID as a random factor. Bonferroni's correction was used in post hoc comparisons between treatments, and *p* < .05 was considered statistically significant for all analyses.

Data from individual animals were removed from analyses if maternal hypoxemia was not achieved or if the blood pressure traces were contaminated with artefacts. The number of animals included in each analysis is described within the relevant Figure legends. Specific details for each treatment group include: ISO‐ *n* = 7, one animal removed from analyses for Figures 4 and 5 as ewe failed to become hypoxic. Another animal removed from analyses for Figure 5 as fetal blood pressure trace was not within the physiological range. ISOKET‐ *n* = 6, one animal removed from analyses for Figures 4 and 5 as ewe failed to become hypoxic, another animal removed from analyses for Figure 5 as MAP dropped from ~26 mmHg at beginning of recording to 10 mmHg during hypoxia, at which point the experiment was terminated. KETMID‐ *n* = 7, all animals tested included in analyses. PRO‐ *n* = 8, all animals tested included in analyses. CON‐ *n* = 9, one animal removed from analyses as both maternal and fetal blood pressure trace compromised (intermittent recording with ~50% fetal data missed).

## 3 RESULTS

### 3.1 Plane of anesthesia

**Figure 2:**
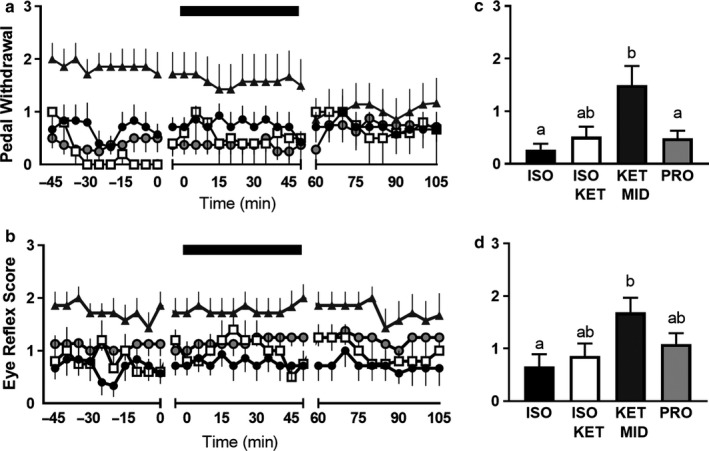
Plane of anesthesia as determined by response to noxious stimuli. Pedal withdrawal (a) and palpebral reflex (b) during periods of baseline recording, hypoxia and recovery in ewes anesthetized with ISO (black circles, *n* = 7), ISOKET (open squares, *n* = 5), KETMID (grey triangles, *n* = 7), or PRO (open circles, *n* = 8). Data are mean ± *SEM*, with time = 0 representing the first recording at which pulse oximeter maternal oxygen saturation dropped and remained below 75%. Black bar represents period of hypoxia; Letters denote significance between treatments; ANOVA with Bonferroni multiple comparisons; *p* < .05

Plane of anesthesia differed between anesthetic regimens, as determined by pedal withdrawal (*p* = .003), and palpebral reflex (*p* = .033, Figure 2), which were higher in ewes administered KET/MID (*p* < .05), indicating a lighter plane of anesthesia.

### 3.2 The impact of anesthetic agent on cardiovascular status during normoxia

**Figure 3:**
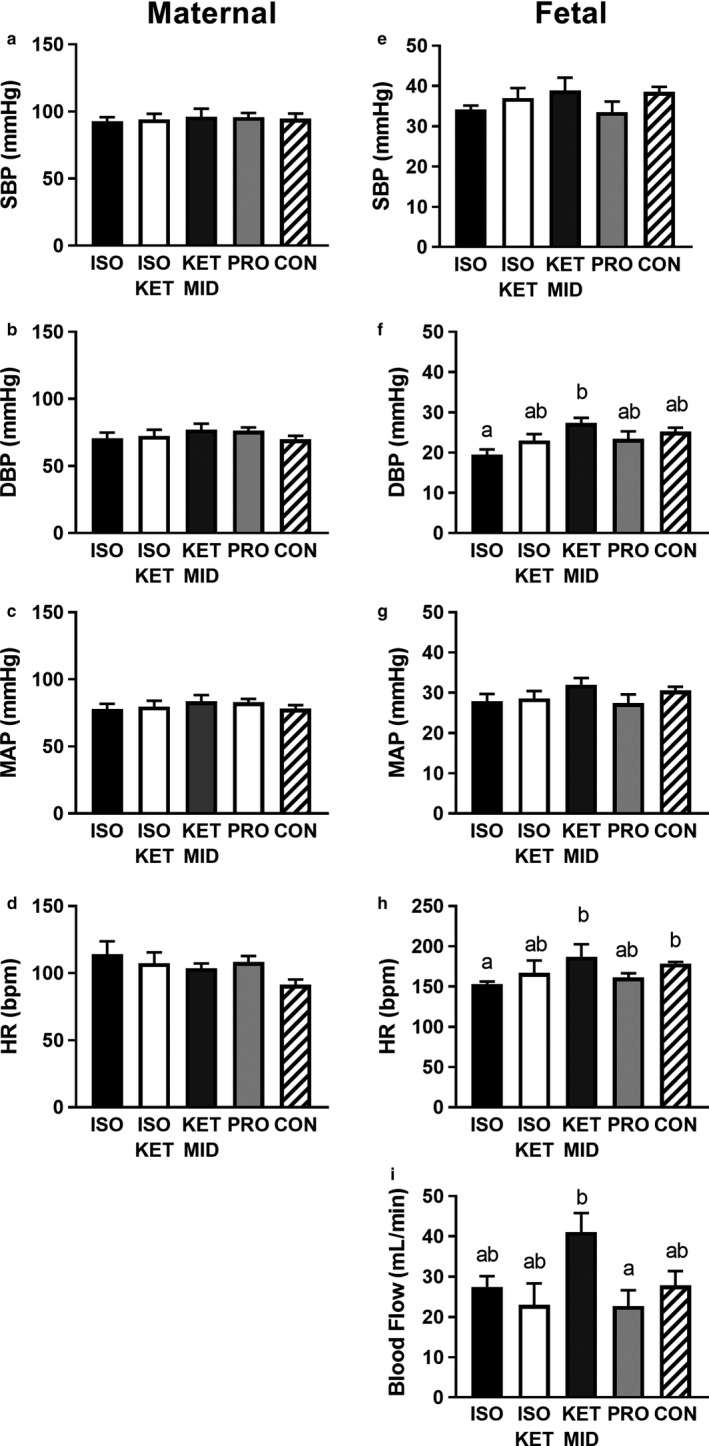
Maternal and fetal cardiovascular status under baseline conditions. Average maternal systolic blood pressure (a), diastolic blood pressure (b), mean arterial pressure (c) and heart rate (d) and fetal systolic blood pressure (e), diastolic blood pressure (f), mean arterial pressure (g), heart rate (h), and peripheral blood flow (i). Data are the average values in the 60 min prior to nitrogen exposure ± *SEM*; ISO, *n* = 6–7; ISO/KET, *n* = 5–6; KET/MID, *n* = 6–7; PRO, *n* = 8; CON, *n* = 7–8; Letters denote significance between treatments; linear mixed model with Bonferroni multiple comparisons, *p* < .05

**Figure 4:**
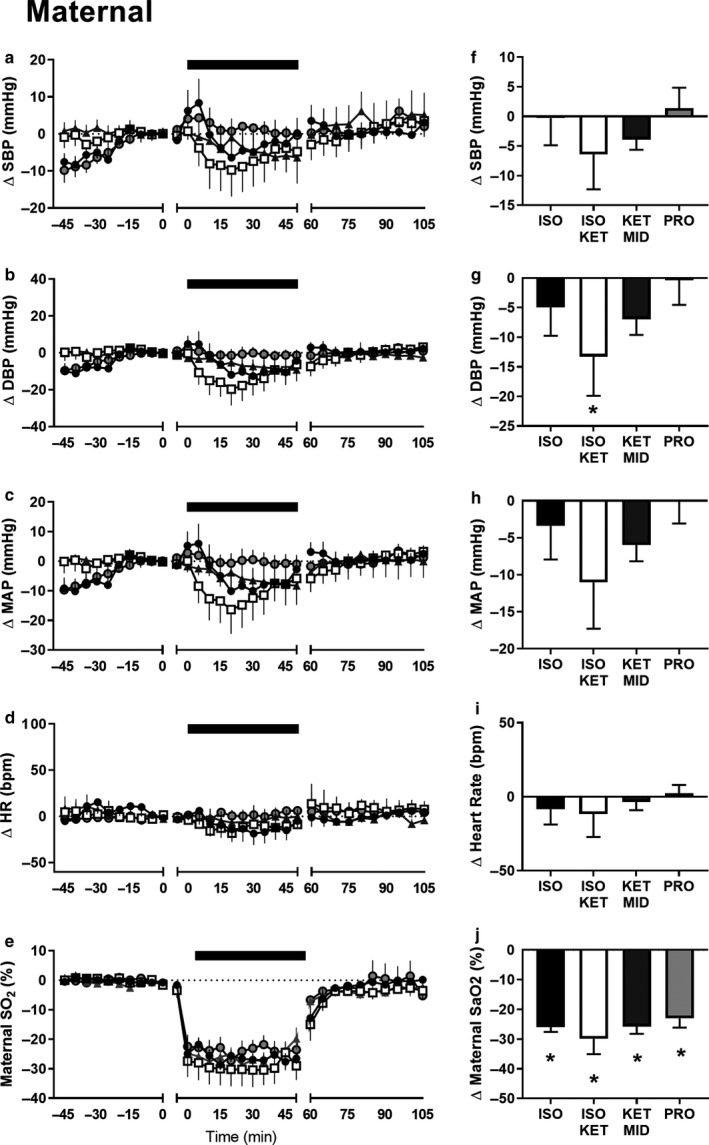
The impact of anesthesia on maternal cardiovascular responses to hypoxia. Maternal systolic blood pressure (a), diastolic blood pressure (b), mean arterial pressure (c), and heart rate (d) during baseline recording (−60 to 0 min, where time = 0 is the time nitrogen was turned on), maternal hypoxia (0 to 60 min, where time = 0 is the first point at which maternal pulse oximetry SaO_2_ dropped below 75%), and recovery (60–105 min, where *n* = 60 is the time at which nitrogen was turned off) in fetuses exposed to ISO (black circles, *n* = 6), ISOKET (clear squares, *n* = 5), KETMID (grey triangles, *n* = 7), or PRO (clear circles, *n* = 8). Black bar represents period of maternal hypoxia. Data are 5‐min average values, expressed relative to the last 10 min of baseline recoding for each animal. The average change in maternal cardiovascular variables during the period of hypoxia (f to i). Maternal oxygen saturation as measured by pulse oximetry during periods of baseline recording and nitrogen exposure (e) and average change during hypoxia (j). Data are the mean ± *SEM*; * denotes a significant change from normoxia; repeated measures linear mixed model with Bonferroni multiple comparisons, *p* < .05

There was no effect of anesthesia upon maternal SBP, DBP, MAP, or HR during the one hour of normoxic baseline recording (Figure 3, *p* > .05). There was no association between anesthesia and fetal SBP or MAP (*p* > .05), but DBP was lower in fetuses exposed to ISO compared with KET/MID (−7.9 ± 2.2, *p* = .022, Figure 4). Heart rate was lower in ISO (−32 ± 8 bpm, *p* = .044) exposed fetuses compared with conscious controls.

### 3.3 The impact of anesthetic agent on maternal and fetal arterial blood gas responses to hypoxia

Anesthesia, regardless of type, reduced maternal PaO_2_ in a time dependent manner (time × treatment, *p* < .001, Table 1). All anesthetics decreased maternal PaO_2_ by ~30 mmHg such that during normoxia and prior to nitrogen exposure, PaO_2_ was lower in ewes administered ISO, ISO/KET, KET/MID, and PRO compared to conscious controls (all *p* < .001). During hypoxia, maternal PaO_2_ in conscious ewes decreased by ~55 mmHg to achieve levels of 51.7 ± 3.6 and 56.2 ± 4.2 mmHg at 15 min and 45 min, respectively (*p* < .001). In anesthetized ewes, PaO_2_ decreased by ~30 mmHg to reach 38.2 ± 1.8 mmHg (ISO), 33.7 ± 4.2 (ISO/KET), 36.0 ± 1.3 mmHg (KET/MID), and 39.5 ± 2.5 mmHg (PRO) 15 min after initiation of nitrogen exposure. Consequently, maternal PaO_2_ in ewes administered anesthesia was approximately 15 mmHg lower than conscious controls during periods of hypoxia (all *p* < .05).

**Table 1:** Maternal arterial blood gas and pH responses to acute hypoxia

		Preanesthesia	Normoxia (−15 min)	Hypoxia (+15 min)	Hypoxia (+45 min)	Recovery (+15 min)	Treat. *p*	Time *p*	Int. *p*
PO_2_ (mmHg)	ISO	99.2 ± 4.2	69.1 ± 3.9^*δ^	38.2 ± 4.2^*δ #^	40.1 ± 4.2^*δ#^	70.6 ± 4.6^*δ^	<.001	<.001	<.001
	ISO/KET	103.4 ± 4.6	69.1 ± 4.6^*δ^	33.7 ± 5.1^*δ#^	34.0 ± 5.1^*δ#^	64.7 ± 5.1^*δ^			
	KET/MID	111.2 ± 4.2	69.4 ± 3.9^*δ^	36.0 ± 3.9^*δ#^	39.4 ± 4.2^*δ#^	72.9 ± 4.6^*δ^			
	PRO	96.9 ± 3.9	65.8 ± 3.6^*δ^	39.5 ± 3.6^*δ#^	40.6 ± 3.6^*δ#^	58.2 ± 4.2^*δ^			
	CON		108.7 ± 3.9	51.7 ± 3.6^#^	56.2 ± 4.2^#^	105.3 ± 3.6			
PCO_2_ (mmHg)	ISO	40.1 ± 2.2	36.4 ± 1.9	31.9 ± 2.2	31.3 ± 2.2	36.4 ± 2.4	.003	<.001	.508
	ISO/KET	38.6 ± 2.4	32.3 ± 2.4	30.5 ± 2.6	27.8 ± 2.6	34.8 ± 2.6			
	KET/MID	32.2 ± 2.2	35.4 ± 2.0	31.5 ± 2.0	31.2 ± 2.2	34.5 ± 2.4			
	PRO	38.2 ± 2.0	40.9 ± 1.9	35.4 ± 1.9	34.3 ± 1.9	34.6 ± 2.2			
	CON		34.6 ± 1.9	32.3 ± 1.9	26.0 ± 2.4	32.6 ± 1.9			
pH	ISO	7.447 ± 0.020	7.441 ± 0.019	7.496 ± 0.020	7.492 ± 0.020	7.421 ± 0.021	.038	<.001	.189
	ISO/KET	7.443 ± 0.021	7.457 ± 0.021	7.526 ± 0.023	7.510 ± 0.023	7.442 ± 0.023			
	KET/MID	7.483 ± 0.020	7.437 ± 0.019	7.474 ± 0.019	7.454 ± 0.020	7.392 ± 0.021			
	PRO	7.463 ± 0.019	7.430 ± 0.018	7.459 ± 0.018	7.457 ± 0.018	7.430 ± 0.020			
	CON		7.457 ± 0.019	7.492 ± 0.018	7.481 ± 0.020	7.467 ± 0.018			
Hb (ml dl^−1^)	ISO	11.2 ± 0.5	8.2 ± 0.5^δ^	8.6 ± 0.5^*δ^	8.4 ± 0.5^δ^	8.3 ± 0.5^δ^	<.001	<.001	.001
	ISO/KET	10.7 ± 0.5	6.9 ± 0.6^*δ^	8.5 ± 0.5^*δ^	8.2 ± 0.6^δ^	8.0 ± 0.6^δ^			
	KET/MID	9.4 ± 0.5	8.7 ± 0.5	9.0 ± 0.5^*^	8.7 ± 0.5	8.9 ± 0.5			
	PRO	10.4 ± 0.5	9.2 ± 0.5	10.2 ± 0.5	10.0 ± 0.5	9.6 ± 0.5			
	CON		9.4 ± 0.5	10.7 ± 0.5	9.4 ± 0.5	9.8 ± 0.5			
SO_2_ (%)	ISO	97.3 ± 4.0	94.0 ± 3.0	59.2 ± 3.3^*δ#^	60.9 ± 3.3^*δ#^	92.1 ± 3.6	<.001	<.001	.018
	ISO/KET	96.7 ± 4.5	96.7 ± 4.0	60.6 ± 4.0^*δ#^	59.6 ± 4.5^*δ#^	93.3 ± 4.0			
	KET/MID	95.1 ± 3.6	91.4 ± 3.0*	57.6 ± 3.0^*δ#^	61.8 ± 3.3^*δ#^	91.9 ± 3.6			
	PRO	97.6 ± 3.6	87.1 ± 3.0*	59.1 ± 3.0^*δ#^	60.2 ± 3.0^*δ#^	84.6 ± 3.3^*^			
	CON		98.3 ± 3.0	79.9 ± 2.9^#^	85.3 ± 3.6^#^	97.0 ± 3.3			

Note

Isoflurane (ISOKET, *n* = 7), isoflurane/ketamine (ISO/KET, *n* = 6), ketamine/midazolam (KET/MID, *n* = 7), propofol (PRO, *n* = 8), and conscious (CON, *n* = 9). Data are the estimated marginal means ± *SEM*. Data are analyzed by repeated measures linear mixed models with Bonferroni post hoc comparisons for interaction effects with *p* < .05 indicated by * for comparisons to CON animals at each time point, by δ for comparisons with preanesthesia, and by ^#^ for comparisons with normoxia.

Compared with conscious controls, maternal PaCO_2_ was higher overall in ewes administered PRO (+ 4.7 ± 1.3 mmHg, *p* = .003), and was lower in all animals 45 min after the initiation of hypoxia (−5.8 ± 1.3 mmHg, *p* < .001). While there was a significant effect of treatment upon maternal pH (*p* = .038), there was no effect of any individual anesthetic (*p* > .05). Hypoxia increased maternal pH at 15 min (+0.045 ± 0.010, *p* < .001) and 45 min (+0.035 ± 0.010, *p* = .011). Total maternal Hb concentrations were higher in ewes administered ISO (+0.8 ± 0.2, *p* = .003), ISO/KET (+1.3 ± 0.2, *p* < .001), and KET/MID (+0.8 ± 0.2, *p* = .006), but not PRO (+0.1 ± 0.2, *p* = 1.0).

There was a time‐dependent effect of anesthesia upon maternal SaO_2_ (time x treatment, *p* = .018). Prior to hypoxia, maternal SaO_2_ was 7.5 ± 2.3% lower in KET/MID and 12.1 ± 2.3% lower in PRO ewes compared to conscious controls (*p* < .05). When exposed to nitrogen, maternal SaO_2_ decreased in all ewes regardless of treatment (*p* < .05) but was 20%–25% lower in ewes administered anesthesia (all *p* < .05).

Anesthesia did not affect fetal PaO_2_ (*p* = .244) or PaCO_2_ (*p* = .128, Table 2). However, hypoxia decreased PaO_2_ at 15 min (−8.5 ± 0.7 mmHg, *p* < .001) and 45 min (−6.7 ± 0.8 mmHg, *p* < .001), and PaCO_2_ at 45 min (−6.7 ± 1.8 mmHg*, p* = .002). pH was lower overall in fetuses exposed to ISO/KET only (−0.039 ± 0.011, *p* = .006). Total fetal hemoglobin was higher overall in ewes administered ISO (+1.5 ± 0.2 ml·dl^−1^, *p* < .001), ISO/KET (+1.7 ± 0.3, *p* < .001), KET/MID (+2.2 ± 0.2, *p* < .001), and PRO (+0.9 ± 0.2, *p* = .002) compared with conscious controls, and increased with hypoxia (+0.8 ± 0.2 ml·dl^−1^, *p* = .014).

**Table 2:** Fetal arterial blood gas and pH responses to acute hypoxia

		Preanesthesia	Normoxia (−15 min)	Hypoxia (+15 min)	Hypoxia (+45 min)	Recovery (+15 min)	Treat. *p*	Time *p*	Int. *p*
PO_2_ (mmHg)	ISO	21.1 ± 1.3	21.1 ± 1.3	12.5 ± 1.7	14.9 ± 1.9	23.1 ± 1.7	.244	<.001	.399
	ISO/KET	22.6 ± 1.4	22.8 ± 1.5	13.0 ± 1.7	14.3 ± 1.7	24.2 ± 1.7			
	KET/MID	22.7 ± 1.5	22.2 ± 1.3	12.7 ± 1.4	14.6 ± 1.3	25.6 ± 1.4			
	PRO	22.2 ± 1.3	20.5 ± 1.3	12.9 ± 1.3	14.0 ± 1.3	19.1 ± 1.5			
	CON		20.9 ± 1.2	14.1 ± 1.2	15.9 ± 1.3	22.2 ± 1.2			
PCO_2_ (mmHg)	ISO	51.3 ± 1.3	50.9 ± 2.7	46.8 ± 2.9	44.1 ± 2.9	44.0 ± 2.9	.128	<.001	.993
	ISO/KET	45.3 ± 2.9	47.3 ± 3.2	40.6 ± 3.6	38.6 ± 3.6	43.5 ± 3.6			
	KET/MID	47.5 ± 2.7	47.0 ± 2.7	43.9 ± 2.7	41.8 ± 2.9	43.6 ± 3.2			
	PRO	51.2 ± 2.5	48.9 ± 2.5	46.1 ± 2.5	43.7 ± 2.5	45.1 ± 2.9			
	CON		47.9 ± 2.5	41.7 ± 2.4	39.0 ± 2.5	47.5 ± 2.4			
pH	ISO	7.378 ± 0.018	7.324 ± 0.018^δ^	7.359 ± 0.020	7.327 ± 0.020	7.323 ± 0.020	<.001	<.001	.026
	ISO/KET	7.346 ± 0.019	7.356 ± 0.021	7.370 ± 0.023	7.320 ± 0.023	7.256 ± 0.023			
	KET/MID	7.396 ± 0.018	7.388 ± 0.018	7.378 ± 0.018	7.346 ± 0.018	7.297 ± 0.020^δ#^			
	PRO	7.384 ± 0.017	7.378 ± 0.017	7.385 ± 0.017	7.392 ± 0.018	7.367 ± 0.020			
	CON		7.359 ± 0.017	7.400 ± 0.017^#^	7.373 ± 0.017	7.355 ± 0.017			
Hb (ml dl^−1^)	ISO	8.9 ± 0.4	8.8 ± 0.4	9.4 ± 0.4	9.5 ± 0.4	8.9 ± 0.5	<.001	.003	.956
	ISO/KET	8.9 ± 0.4	8.7 ± 0.4	9.9 ± 0.5	9.7 ± 0.5	9.3 ± 0.5			
	KET/MID	9.0 ± 0.7	9.5 ± 0.4	10.3 ± 0.4	10.2 ± 0.4	9.9 ± 0.4			
	PRO	8.3 ± 0.4	8.3 ± 0.4	8.7 ± 0.4	8.8 ± 0.4	8.3 ± 0.4			
	CON		7.4 ± 0.3	8.1 ± 0.3	7.3 ± 0.4	7.7 ± 0.3			
SO_2_ (%)	ISO	63.4 ± 3.5	52.8 ± 3.5^δ^	18.1 ± 3.8^*δ#^	22.9 ± 3.8^*δ#^	56.6 ± 4.1	<.001	<.001	.034
	ISO/KET	55.3 ± 3.9	57.0 ± 3.5	25.2 ± 5.2^δ#^	14.4 ± 6.5^*δ#^	55.5 ± 5.2			
	KET/MID	61.2 ± 3.5	57.3 ± 3.5	23.7 ± 3.8^δ#^	27.2 ± 3.5^δ#^	60.1 ± 3.8			
	PRO	62.9 ± 3.3	59.8 ± 3.5	30.8 ± 3.8^δ#^	33.9 ± 4.1^δ#^	50.4 ± 4.1			
	CON		61.1 ± 3.2	36.6 ± 3.2^#^	40.1 ± 3.3^#^	64.3 ± 3.2			

Note

Isoflurane (ISOKET, *n* = 7), isoflurane/ketamine (ISO/KET, *n* = 6), ketamine/midazolam (KET/MID, *n* = 7), propofol (PRO, *n* = 8), and conscious (CON, *n* = 9). Data are the estimated marginal means ± *SEM*. Data are analyzed by repeated measures linear mixed models with Bonferroni post hoc comparisons for interaction effects with *p* < .05 indicated by * for comparisons to CON animals at each time point, by ^δ^ for comparisons with preanesthesia, and by ^#^ for comparisons with normoxia.

Hypoxia reduced fetal SaO_2_ in a time dependent manner (time x treatment interaction, *p* = .034). While fetal SaO_2_ decreased in all animals at 15 min (−33.9 ± 2.2, *p* < .001) and 45 min (−33.1 ± 2.2, *p* < .001) following the initiation of hypoxia, ISO, and ISO/KET fetuses were significantly more hypoxic than conscious controls (*p* < .05).

### 3.4 The impact of anesthetic agent on maternal and fetal cardiovascular responses to hypoxia

**Figure 5:**
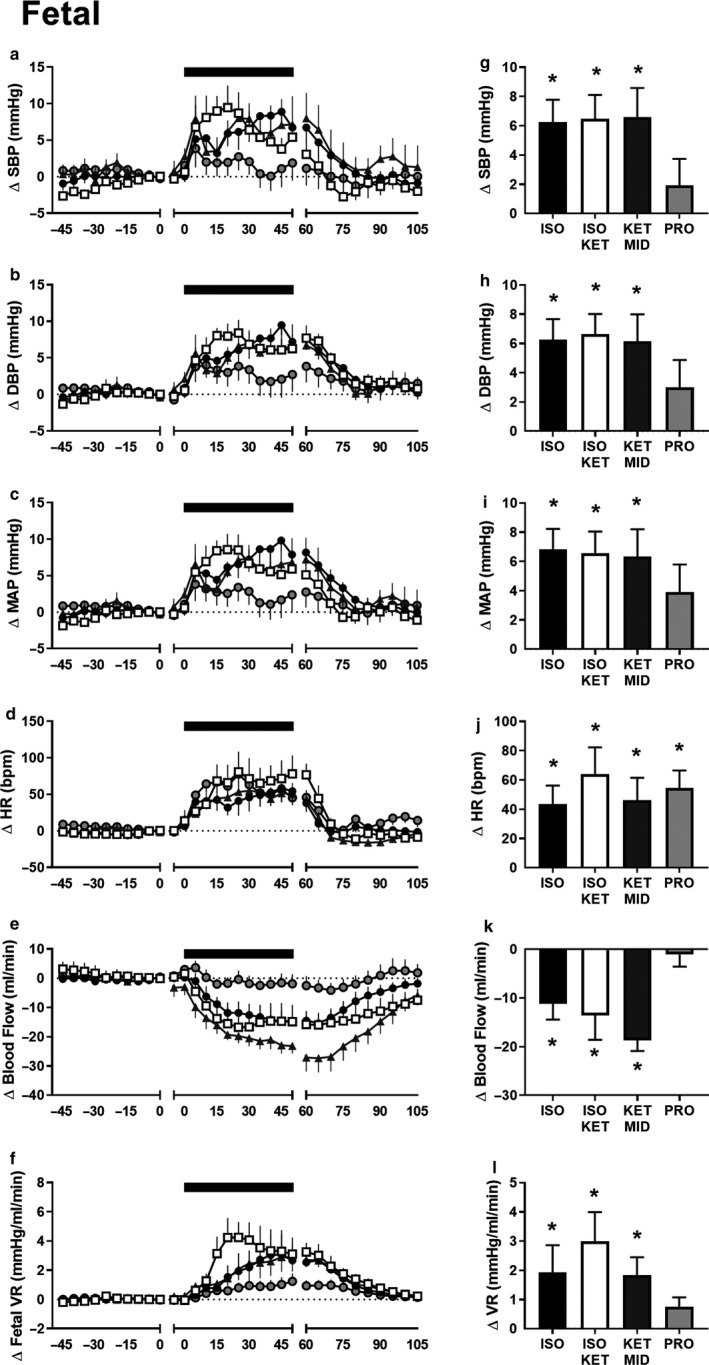
The impact of anesthesia on fetal cardiovascular responses to maternal hypoxia. Fetal systolic blood pressure (a), diastolic blood pressure (b), mean arterial pressure (c), heart rate (d), femoral blood flow (e), and vascular resistance (f) during baseline recording (−60 to 0 min, where time = 0 is the time nitrogen was turned on), maternal hypoxia (0 to 60 min, where time = 0 is the first point at which maternal pulse oximetry SaO_2_ dropped below 75%), and recovery (60 to 105 min, where *n* = 60 is the time at which nitrogen was turned off) in fetuses exposed to ISO (black circles, *n* = 5), ISOKET (clear squares, *n* = 4), KETMID (grey triangles, *n* = 7) or PRO (clear circles, *n* = 8). Black bar represents period of maternal hypoxia. Data are 5‐min average values, expressed relative to the last 10 min of baseline recoding for each animal. The average change in fetal cardiovascular variables during the period of hypoxia (g to l). Data are the mean ± *SEM*; * denotes a significant change from normoxia; repeated measures linear mixed model with Bonferroni multiple comparisons, *p* < .05

Exposure to hypoxia had no effect on maternal MAP, SBP, or heart rate (*p* > .05), but decreased DBP in ewes administered ISO/KET (*p* = .025, Figure 4). Exposure to hypoxia increased fetal MAP, SBP, DBP, VR, and decreased femoral blood flow in fetuses exposed to ISO, ISO/KET, and KET/MID (*p* < .05), but not PRO (*p* > .05, Figure 5). In comparison, hypoxia increased fetal heart rate, regardless of the anesthetic agent used (*p* < .05).

## 4 DISCUSSION

To identify the most appropriate anesthetic regime for preclinical MRI studies of fetal hemodynamics, we compared the maternal and fetal blood gas and cardiovascular response to four anesthetic drugs commonly used in veterinary medicine (Flecknell, 2015; Riebold et al., 2015). The results demonstrate that anesthetic agents have differential effects upon maternal and fetal hemodynamics during normoxia and in response to an acute hypoxic challenge. During normoxia, all anesthetic regimes tested in this study induced maternal hypoxemia, whereas only isoflurane decreased fetal heart rate. When exposed to acute hypoxia, the expected fetal cardiovascular response of increased blood pressure, reduced peripheral blood flow, and increased vascular resistance was observed in all fetuses except those administered propofol. Hypoxia also increased fetal heart rate, regardless of the anesthetic regime, indicating a change in the fetal response to periods of acute hypoxia when exposed to maternal anesthesia compared to when no anesthesia is present.

A well‐known side effect of anesthesia is cardiovascular depression, characterized by a reduction in cardiac output, systemic vascular resistance, and mean arterial pressure (Claeys, Gepts, & Camu, 1988; Khan et al., 2014). These cardiovascular responses have previously been observed in pregnant ewes, with mean arterial pressure dropping by up to 40% during propofol or isoflurane anesthesia (Andaluz, Trasserras, & García, 2005; McClaine et al., 2005; Palahniuk & Shnider, 1974). Alternatively, a single bolus dose of midazolam (0.5 mg kg^−1^) or ketamine (5 mg kg^−1^) to pregnant ewes increased maternal blood pressure (Conklin et al., 1980; Levinson, Shnider, Gildea, & DeLorimier, 1973). In our study, basal mean arterial pressure was not affected by any of the anesthetics tested. This may be due to anesthesia‐induced hypoxemia, as we ventilated the ewes with air rather than 100% oxygen to avoid hyperoxygenation of the fetus. Consequently, maternal PaO_2_ was approximately 70 mmHg in ewes under anesthesia, compared to ~100 mmHg when conscious. This in turn may have induced a sympathetic compensatory response whereby blood pressure increased, preventing the previously observed decline.

Despite maternal hypoxemia, there was no effect of anesthesia upon fetal PaO_2_ or PaCO_2_, and pH was lower only in fetuses from isoflurane/ketamine administered ewes. However, fetal hemoglobin was higher overall in anesthetized fetuses. Fetal hemoglobin levels increase in response to acute hypoxemia (Fletcher, Gardner, Edwards, Fowden, & Giussani, 2003), and may have increased here in response to low fetal oxygen saturation during hypoxia. Alternatively, total hemoglobin may have increased in direct response to the anesthetic agents. Indeed, maternal anesthesia with isoflurane can lead to increased total hemoglobin levels in the fetus, even when the ewe was supplemented with oxygen and fetal oxygen saturation was high (McClaine et al., 2005).

Fetal arterial pressure was not affected by anesthesia during the 60 min of baseline recording; however, heart rate was lower in fetuses from ewes anesthetized with isoflurane. Previous studies have demonstrated that with increasing doses of isoflurane, both fetal blood pressure and heart rate decrease (Palahniuk & Shnider, 1974). Alternatively, propofol infusion has been found to increase fetal heart rate and arterial blood pressure (Andaluz et al., 2005). Importantly in both of these studies, ewes were supplemented with 100% oxygen, which in turn increased maternal PaO_2_ to more than 200 mmHg, with corresponding increases in fetal PaO_2_, PaCO_2,_ and a decrease in pH (Andaluz et al., 2005; Palahniuk & Shnider, 1974). As hyperoxyemia itself alters fetal cardiovascular function (Almström & Sonesson, 1996; Khatib et al., 2018; Sonesson, Fouron, Teyssier, & Bonnin, 1994), the fetal response to anesthesia in these studies is not comparable to the results presented here.

In our study, maternal hypoxia was induced by displacement of oxygen with nitrogen into the maternal trachea. While effective when the ewes were immobilized by anesthesia and ventilated at a set respiratory rate, this approach was less effective in conscious animals, as they were able to cough or increase respiratory rate/depth to displace the nitrogen from their airways. While it was not possible to continuously monitor SaO_2_ through pulse oximetry while the ewe was moving, we suspect that the level of hypoxemia fluctuated throughout the 60 min of nitrogen administration. Consequently, while the maternal PaO_2_ decreased from approximately 110 mmHg to 54 mmHg in conscious ewes, maternal oxygen saturation dropped to only 83%, which was significantly higher than that of anesthetized animals and well above the target of less than 75%. These animals are therefore not appropriate controls and limit our capacity to compare the fetal cardiovascular response. We therefore rely on the previously well documented fetal hypoxic response (Bennet, 2017; Boddy et al., 1974; Cohn et al., 1974; Giussani, 2016; Giussani et al., 1993; Raff & Wood, 1992; Wassink et al., 2007), although we recognize this as a limitation of the study.

During periods of acute hypoxia, the fetus employs compensatory mechanisms that prioritize oxygenated blood flow to the brain, heart, and adrenal at the expense of peripheral tissues (Giussani, 2016; Morrison, 2008) reviewedin. Bradycardia, peripheral vasoconstriction, and elevated mean arterial pressure are central to this response and have been demonstrated in fetal sheep following maternal ventilatory hypoxia and umbilical cord occlusion (Boddy et al., 1974; Cohn et al., 1974; Giussani et al., 1993; Raff & Wood, 1992; Wassink et al., 2007). In our study, maternal nitrogen exposure caused fetal PaO_2_ to drop ~7 mmHg, which in turn was followed by increased mean arterial blood pressure, decreased peripheral blood flow and hence increased peripheral vascular resistance in fetuses exposed to isoflurane, isoflurane/ketamine, and ketamine/midazolam. This indicates that the fetal cardiovascular response to hypoxia was maintained during anesthesia. In contrast, fetuses exposed to propofol demonstrated a blunted mean arterial pressure and peripheral blood flow response to acute hypoxia. Propofol impairs vascular reactivity by decreasing sympathetic vasoconstrictor nerve activity (Robinson, Ebert, O’Brien, Colinco, & Muzi, 1997), and by reducing noradrenaline‐induced constriction of arterial endothelial cells (Boillot, Laurant, Berthelot, & Barale, 1999). Given the fetal peripheral vasoconstriction response to acute hypoxia is mediated in large part by the sympathetic nervous system (Booth et al., 2012; Iwamoto, Rudolph, Mirkin, & Keil, 1983; Lewis, Donovan, & Platzker, 1980; Robillard, Nakamura, & DiBona, 1986), the attenuated blood pressure response during propofol anesthesia was not surprising.

Regardless of the type of anesthetic agent administered, fetal heart rate increased in response to hypoxia. This response is not consistent with the expected hypoxia‐induced bradycardia previously demonstrated in conscious sheep (Boddy et al., 1974; Cohn et al., 1974; Giussani et al., 1993; Raff & Wood, 1992). However, in all of these studies, maternal and hence fetal hypoxemia was induced while supplementing CO_2_ in the inspired gas. Alternatively, in our study, by displacement of oxygen with nitrogen directly into the trachea, maternal PaCO_2_, was lower during the period of hypoxia. Using a similar approach, Chang and Wood (2015) demonstrated that displacing oxygen with nitrogen without CO_2_ supplementation does not induce a bradycardic response in the fetus. They did not; however, observe the elevation in heart rate that was consistently observed upon the initiation of hypoxia in our study.

Previous studies have demonstrated that the fetal heart rate response to hypoxia is affected by anesthesia. In fetal lambs exteriorized and anesthetized with sodium pentobarbitone, maternal hypoxemia induced a tachycardic response in the fetus (Parker & Purves, 1967). Similarly, under general maternal anesthesia, reducing oxygen concentration in the inspired gas mixture increased fetal arterial pressure, decreased peripheral blood flow, and induced tachycardia (Dawes et al., 1969). Ketamine infusion directly to the fetus attenuates the fetal bradycardic response to both umbilical cord occlusion (Boekkooi, Baan, Teitel, & Rudolph, 1995; Zarate, Chang, Antolic, & Wood, 2016), and maternal ventilatory hypoxia (Chang & Wood, 2015), whereas maternal ketamine anesthesia following umbilical cord occlusion has also been found to increase fetal heart rate by approximately 55 bpm (Swartz, Cumming, & Biehl, 1987). Together these results demonstrate that the fetal heart rate response to changing PaO_2_ is influenced by maternal and/or fetal anesthesia. Strikingly, the fetal heart rate response to acute hypoxia was very consistent between the anesthetics. Given the pharmacodynamics of each of the anesthetic combinations tested in this study differ, the mechanisms by which they alter the fetal heart rate response to hypoxia is likely to be as a consequence of shared pathway downstream of general anesthesia rather than direct effects upon the relevant receptors. Interestingly, the heart rate profile of fetuses exposed to hypoxia under anesthesia is similar to that when vagal inputs are suppressed by pretreatment with the muscarinic agonist atropine (Giussani et al., 1993), and may indicate that anesthesia alters the autonomic balance in favor of a sympathetic response. Nevertheless, these results suggest that the fetal cardiovascular response to acute hypoxia is altered by maternal anesthesia, although an appropriate conscious control that achieves the same level of hypoxemia is required to confirm this assertion. Certainly, the developing fetus has a variety of strategies in place to ensure appropriate tissue oxygenation throughout development (Giussani, 2016). In the face of acute hypoxia, the normal fetal response is aimed at decreasing oxygen consumption. The heart is a major metabolic organ and although decreasing heart rate aligns with this strategy, it also allows for an increase in end diastolic volume and filling pressure, thus improving stroke volume and maintaining cardiac output (Anderson, Glick, Killam, & Mainwaring, 1986). Given that the normal bradycardic response may be impaired by anesthesia, this has implications for the interpretation of hemodynamic MRI studies of fetal blood flow where anesthesia is necessary to immobilize the ewe.

By administering four different anesthetic regimes to pregnant ewes, we aimed to identify the most appropriate anesthetic for preclinical studies examining fetal hemodynamics. Unfortunately, we were not able to achieve a similar depth of anesthesia between the experimental conditions. While ewes were continuously monitored throughout the protocol for their response to pedal withdrawal and palpebral reflex and the dose of anesthesia adjusted accordingly, ewes administered ketamine/midazolam were more reactive to the stimuli, indicative of a shallower depth of anesthesia. This may limit our capacity to directly compare hemodynamic responses between anesthetic agents.

In conclusion, we have demonstrated that components of the fetal hemodynamic response to an acute hypoxic challenge are maintained during maternal isoflurane, isoflurane/ketamine, and ketamine/midazolam anesthesia, but not during propofol anesthesia. Specifically, mean arterial pressure increased and peripheral blood flow decreased in response to fetal hypoxemia. However, the well‐characterized response of fetal bradycardia was not observed, rather fetal hypoxemia induced tachycardia, regardless of the anesthetic regime. Since the classical bradycardic response aims to maintain cardiac output and perfusion pressure, future studies would be well placed to determine whether the anesthesia‐induced hypoxic tachycardia either detrimentally increased oxygen consumption and/or decreased oxygen delivery to vascular beds. Although the mechanism by which anesthesia impaired the fetal hypoxic response was not identified in this study, these results have important implications for the interpretation of preclinical imaging studies using technologies such as MRI for which anesthesia is required.

## CONFLICT OF INTEREST

The authors have no conflict of interest.

## AUTHOR CONTRIBUTIONS

JLM conceptualized designed the work. JRTD, JLM, TJV, SLH, and ELB acquired or analyzed or interpreted the data for the work. TJV, JLM, JRTD, LV, TRK, SLH, ELB, MW, and MS drafted the work or revised it critically for important intellectual content. JLM, JRTD, TJV, LV, TRK, SLH, ELB, MW, and MS finally approved the version to be published. All the authors agreed to be accountable for all aspects of the work.
